# Supplementary material for: Salivary Biomarkers in COVID-19 Patients: Towards a Wide-Scale Test for Monitoring Disease Activity
Source: J Pers Med. 2021 May 8;11(5):385. doi: 10.3390/jpm11050385 (PMC8151878; doi:10.3390/jpm11050385)
Supplement: Supplementary file 1 [file jpm-11-00385-s001.zip › jpm-1202051-supplementary.pdf]

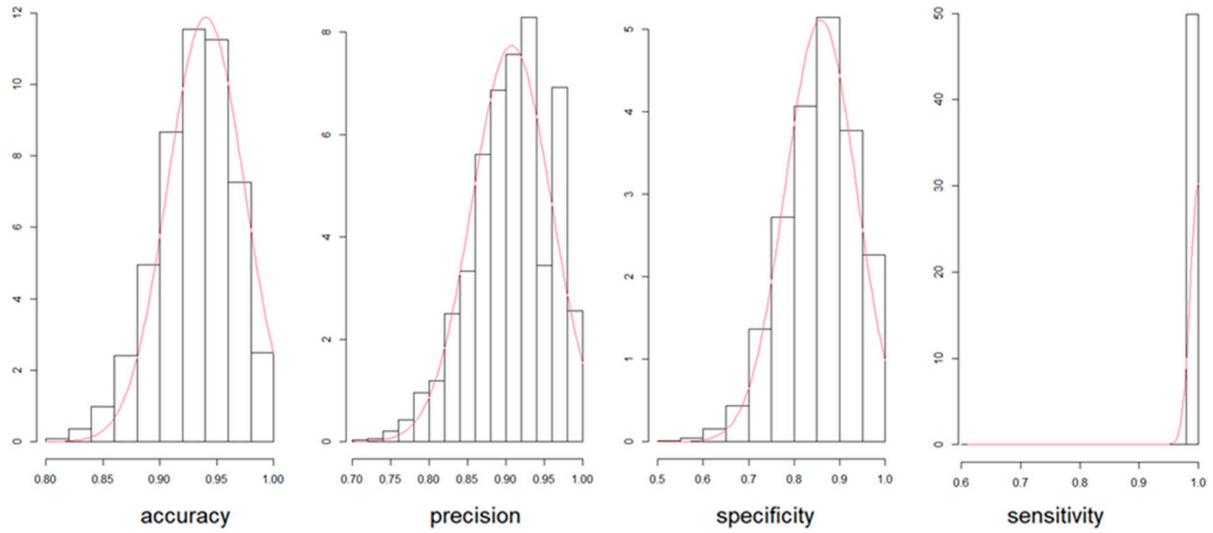

Figure S1: Gaussian fits of the distribution in figure 3 (A-D) used to computed mean accuracy, precision, specificity, and sensitivity together with the corresponding confidence intervals.

		WITHOUT			WITH			
		n	Median	IQR	n	Median	IQR	p-value
Diabetes	k	26	2.49	2.98	3	0.64	0.93	0.15
	l	26	0.78	1.77	3	0.70	1.79	0.97
	k/l	26	1.75	1.94	3	0.50	0.26	0.04
	k+l	26	3.93	5.13	3	1.34	2.72	0.32
	IgA1	26	143.45	366.33	3	36.30	121.35	0.12
	IgA2	26	121.65	254.45	3	34.10	78.60	0.15
Hypertension	k	23	2.38	3.02	6	1.38	2.11	0.13
	l	23	0.70	1.77	6	0.90	2.57	0.75
	k/l	23	1.96	2.03	6	0.57	0.43	0.02
	k+l	23	3.43	5.35	6	2.89	4.85	0.31
	IgA1	23	153.80	326.40	6	85.25	185.45	0.40
	IgA2	23	124.50	246.25	6	82.00	121.68	0.45
Obesity	k	25	2.12	3.12	4	2.51	2.77	0.97
	l	25	0.70	1.77	4	0.90	0.77	0.81
	k/l	25	1.35	2.15	4	2.42	0.68	0.64
	k+l	25	3.43	5.09	4	3.40	3.54	0.97
	IgA1	25	153.80	415.70	4	83.10	81.33	0.64
	IgA2	25	124.50	268.90	4	101.75	69.23	0.65
Oncology history	k	27	2.05	2.97	2	8.82	4.72	0.08
	l	27	0.70	1.84	2	0.94	0.24	0.75
	k/l	27	1.35	1.92	2	11.42	7.92	0.04
	k+l	27	3.08	5.08	2	9.76	4.49	0.18
	IgA1	27	95.40	245.05	2	1564.65	1276.95	0.11
	IgA2	27	118.80	221.15	2	200.85	155.15	0.90
Heart disease	k	25	2.38	3.12	4	1.89	0.83	0.55
	l	25	0.70	1.76	4	2.35	3.37	0.47
	k/l	25	1.54	1.96	4	0.78	0.67	0.15
	k+l	25	3.43	5.33	4	4.38	4.34	0.97
	IgA1	25	133.10	242.20	4	173.80	356.85	0.92
	IgA2	25	118.80	227.20	4	139.00	212.63	0.93
Fever at onset	k	7	2.05	1.58	22	2.49	3.22	0.66
	l	7	0.70	1.04	22	0.90	1.77	0.76
	k/l	7	2.46	1.88	22	1.35	2.16	0.94
	k+l	7	2.75	3.45	22	3.94	5.37	0.70
	IgA1	7	133.10	170.65	22	124.60	435.15	0.94
	IgA2	7	148.90	131.65	22	101.75	292.08	0.50
		n	Median	IQR	n	Median	IQR	p-value
		WITHOUT			WITH			

WITHOUT			WITH					
n	Median	IQR	n	Median	IQR	p-value		
22	1.85	2.64	7	3.82	0.90	0.1	k	Cough at onset
22	0.70	0.69	7	2.47	2.04	0.06	l	
22	1.45	2.10	7	1.35	2.15	0.46	k/l	
22	2.59	4.70	7	5.34	2.18	0.12	k+l	
22	83.80	221.33	7	294.00	618.90	0.09	IgA1	
22	83.70	214.78	7	249.10	435.10	0.16	IgA2	
22	2.09	2.56	7	3.82	8.10	0.61	k	Dyspnea at onset
22	0.78	1.77	7	0.70	1.60	0.89	l	
22	1.45	2.04	7	1.12	3.37	0.70	k/l	
22	3.26	4.49	7	5.10	9.41	0.66	k+l	
22	143.45	228.83	7	60.50	774.00	0.82	IgA1	
22	136.70	214.05	7	45.70	360.95	0.26	IgA2	
19	2.59	2.64	10	1.31	2.60	0.18	k	Asthenia at onset
19	1.17	2.25	10	0.70	0.29	0.14	l	
19	1.54	2.18	10	1.32	1.75	0.46	k/l	
19	5.10	4.85	10	2.01	2.90	0.13	k+l	
19	232.10	602.40	10	85.25	107.03	0.15	IgA1	
19	124.50	298.20	10	82.00	189.93	0.18	IgA2	
8	2.09	2.16	21	2.38	3.28	0.85	k	Pneumonia
8	0.78	2.55	21	0.70	1.76	0.66	l	
8	1.90	1.94	21	1.35	2.17	0.90	k/l	
8	3.59	4.32	21	3.43	5.25	0.79	k+l	
8	182.60	196.60	21	95.40	429.30	0.51	IgA1	
8	164.65	155.73	21	97.60	243.00	0.30	IgA2	
18	1.85	3.09	11	3.35	2.14	0.21	k	Oxygen therapy
18	0.70	2.14	11	0.86	0.48	0.72	l	
18	1.24	1.80	11	3.07	2.23	0.03	k/l	
18	2.59	5.57	11	4.44	2.88	0.49	k+l	
18	112.80	235.13	11	153.80	671.85	0.87	IgA1	
18	136.70	214.18	11	105.90	253.85	0.71	IgA2	
23	2.38	3.02	6	1.18	1.88	0.81	k	Smoke
23	1.09	1.84	6	0.70	0.12	0.72	l	
23	1.54	1.93	6	0.78	1.44	0.29	k/l	
23	4.44	5.35	6	1.88	2.73	0.99	k+l	
23	153.80	326.40	6	96.80	165.00	0.96	IgA1	
23	118.80	218.50	6	99.45	238.33	0.87	IgA2	
n	Median	IQR	n	Median	IQR	p-value		
WITHOUT			WITH					

Table S1: Effect of comorbidities, symptoms at onset, complications and smoke on selected salivary biomarker levels.
